# FGF18 Inhibits Clear Cell Renal Cell Carcinoma Proliferation and Invasion via Regulating Epithelial-Mesenchymal Transition

**DOI:** 10.3389/fonc.2020.01685

**Published:** 2020-09-29

**Authors:** Chen Yang, Zheyu Zhang, Fangdie Ye, Zezhong Mou, Xinan Chen, Yuxi Ou, Chenyang Xu, Siqi Wu, Zhang Cheng, Jimeng Hu, Lujia Zou, Haowen Jiang

**Affiliations:** ^1^Department of Urology, Huashan Hospital, Fudan University, Shanghai, China; ^2^Fudan Institute of Urology, Huashan Hospital, Fudan University, Shanghai, China; ^3^National Clinical Research Center for Aging and Medicine, Fudan University, Shanghai, China

**Keywords:** FGF18, epithelial-mesenchymal transition, clear cell renal cell carcinoma, proliferation, invasion

## Abstract

Fibroblast growth factor 18 (FGF18) is a member of the FGF family and contributes to a broad range of biological events. The important role of the overexpression of FGF18 has been identified in the progression of several types of cancers. However, there is still little information on the biological role of FGF18 on clear cell renal cell carcinoma (ccRCC), which is of interest in investigating the biological functions of FGF18 in ccRCC. Our results showed that FGF18 was lowly expressed in ccRCC tissues compared to paired normal renal tissue from the TCGA database and clinical cohort of Huashan Hospital and that high expression of FGF18 correlated with a good prognosis in ccRCC patients. In addition, overexpression of FGF18 significantly inhibited the proliferation ability of ccRCC cell lines *in vitro* and *in vivo*. Gene set enrichment analysis (GSEA) identified epithelial-mesenchymal transition (EMT) involved in a high FGF18 expression group of ccRCC patients in the TCGA cohort, which was further validated with EMT related markers in FGF18 overexpressed ccRCC cell lines. Furthermore, FGF18 overexpression significantly inhibited the PI3K/Akt pathway in ccRCC cells. Taken together, this study concludes that FGF18 is of potential value as a target for ccRCC.

## Introduction

Renal cell carcinoma (RCC) caused an estimated 65,340 new cases in 2018 and is the sixth most diagnosed neoplasm in men and tenth in women (1). Clear cell renal cell carcinoma (ccRCC) or kidney renal clear cell carcinoma (KIRC) accounts for 70–85% histologic subtypes of RCC, which derives from the tubule epithelium of renal parenchyma (2). In its early stages, ccRCC lacks conspicuous symptoms and approximately 25–30% of patients eventually suffer from metastatic RCC (mRCC) (3). Unfortunately, approximately 40% of patients are resistant to conventional chemotherapy and radiation therapy. Systemic recurrence may occur in some patients, while mRCC patients who have experienced treatment failure have a 5-year survival rate of less than 20% (4). Somatic mutant genes in ccRCC are identified with whole genome sequencing and have been linked with pathogenesis and the mechanisms of ccRCC (5). However, there is an urgent need for further methods of recognizing these crucial biomarkers to facilitate early detection and counter the devastating progression of ccRCC.

Fibroblast growth factor receptors (FGFRs) are highly conserved transmembrane tyrosine kinase receptors (TKRs) among humans, mice, and rats. The FGF-FGFR pathway contributes to a broad range of biological events, including tissue development, angiogenesis, regeneration, and tumorigenesis. Fibroblast growth factor 18 (FGF18), a 21.2 kDa glycosylated secretory protein, is essential for the embryonic and postnatal development of cartilage, hair, and vasculature (6, 7). Overexpression of FGF18 has been frequently identified in several neoplasms, including hepatocellular carcinoma (8), gastric cancer (9), and colon cancer (10). FGF18 is also a prognostic and therapeutic biomarker for certain types of ovarian cancers (11), while its underlying pathophysiological role in ccRCC progression remains elusive.

In this work, we comprehensively revealed the differential expression and clinical correlation of FGF18 in ccRCC. In contrast, less expression of FGF18 has been observed both in the TCGA database, clinical cohort, and cell lines of ccRCC. We further investigated whether the activation of FGF18 inhibits renal carcinogenesis by regulating epithelial-mesenchymal transition (EMT), which aimed to identify FGF18 as novel prognostic biomarkers and therapeutic targets for clinical intervention of ccRCC.

## Materials and Methods

### Study Approval

The study protocol were approved by the Ethics Committee of Huashan Hospital (Shanghai, China; approval no. KY2011-009) and conducted in accordance with the tenets of the Declaration of Helsinki. All patients consented to the use of resected tissues for research purposes.

### Computational Analysis of FGF18 in Several Cancer Tissues

Gene expression profiling were analyzed by using UALCAN^1^ (12). In the UALCAN database, we identified cancer types including, BRCA (Breast invasive carcinoma), BLCA (Bladder Urothelial Carcinoma), COAD (Colon adenocarcinoma), LUAD (Lung adenocarcinoma), KIRC (Kidney renal clear cell carcinoma), PAAD (Pancreatic adenocarcinoma), PRAD (Prostate adenocarcinoma), LIHC (Liver hepatocellular carcinoma), and STAD (Stomach adenocarcinoma). These types were selected and differentially expressed FGF18 were downloaded. Next, according to the average expression level of FGF18, KIRC patients from the Cancer Genome Atlas database were divided into an FGF18 high expression group and an FGF18 medium/low expression group.

### Total RNA Isolation and qRT-PCR

Total RNA was isolated from the tumor tissues or cells using TRIzol Reagent (Invitrogen) following the manufacturer’s protocol. The purity and concentration of the RNA samples were examined by measuring the absorbance at 260, 280, and 230 nm using the NanoDrop ND-100 (Thermo Fisher Scientific). Specifically, optical density (OD)260/OD280 ratios between 1.8 and 2.1 were deemed acceptable, and OD260/OD230 ratios greater than 1.8 were deemed acceptable.

RNA was reverse transcribed into cDNA using SuperScript II Reverse Transcriptase (Invitrogen). Then qRT-PCR was performed using an AB7300 thermo-recycler (Applied Biosystems) with primers (Gene Pharma) and the TaqMan Universal PCR Master Mix. GAPDH was used as the reference gene for FGF18 mRNA.

The primers used to value FGF18 expression were:

Forward, 5-TGCTTCCAGGTACAGGTGCT-3;

Reverse, 5-GCTGCTTACGGCTCACATCG-3.

The GAPDH primers were:

Forward, 5-GCACCGTCAAGGCTGAGAAC-3;

Reverse, 5-GGATCTCGCTCCTGGAAGATG-3.

### Cell Culture and Reagents

HEK293T, Human normal renal tubular epithelial cell line HK-2 and Human renal cancer cell lines (769-P and A498) were purchased from Shanghai Yuanye. Human renal cancer cell lines (Caki and 786-O) were purchased from the Institute of Biochemistry and Cell Biology, Chinese Academy of Sciences. HK2, A498, 786-O were cultured in DMEM (Gibco), and Caki-1 was cultured in McCoy’s medium (Gibco). 769-P was cultured in RPMI 1640 medium (Gibco) and supplemented with 10% fetal bovine serum (Gibco) and 100 units/mL penicillin-streptomycin (Gibco). Antibodies including anti-rabbit IgG-HRP and anti-mouse IgG-HRP horseradish peroxidase were purchased from Jackson ImmunoResearch. The antibodies anti-FGF18 for Slug and Snail were purchased from Cell Signaling Technology. Antibodies including anti-E-cadherin, N-Cadherin, p-PI3K, PI3K, p-AKT, and AKT were purchased from Abcam. The antibodies GAPDH and vimentin were purchased from Proteintech. Dimethyl sulfoxide (DMSO), CCK-8 was obtained from Sigma-Aldrich. LY294002 was obtained from Selleck and recombinant FGF18 protein was obtained from Sino Biological.

### FGF18 Lentiviral Packaging and Infection

To overexpress FGF18, the recombinant plasmid vector pENTER encoding FGF18 protein, and NC were purchased from Vigene (MM_003862). HEK293T cells were seeded into 10 cm culture dish 24 h before transfection with PSPAX2 and PMD2G. A cell medium containing virus particles was collected at 72 h post-transfection. After filtration (0.45 μm filter), the cell medium was added to the 786-O and Caki-1 for viral infection with fresh medium replenished 24 h later. 1.0 μg/ml puromycin (Sangon) selection was started at 48 h post-infection. The protein of FGF18 expression was detected by Western blotting analysis.

### Cell Viability Assay

Cell Counting Kit-8 (CCK8) assay was used to detect cellular proliferation. The transfected cells were seeded into 96-well plates at a density of 2500 Caki-1 and 786-O cells per well in triplicate wells. Cell viability was measured at 0, 24, 48, and 72 h after seeding, according to the manufacturer’s instructions.

### Colony Formation Assay

Caki and 786-O cells were seeded at 600 cells per well in six-well plates. Cells were allowed to grow for 8 or 9 days and stained with crystal violet solution to assess colony growth. The colonies were imaged and counted after they were fixed with 4% paraformaldehyde and stained.

### Transwell Assay

Cell migration was analyzed using Transwell chambers (Corning) in accordance with the manufacturer’s protocol. After incubation for 24 h, the cells on the upper surfaces of the Transwell chambers were removed and the cells located on the lower surfaces were fixed with 4% paraformaldehyde for 10 min, followed with crystal violet staining. The stained cells were photographed and counted in five randomly selected fields.

### Western Blot Analysis

Lysates from cells and tumor tissues were prepared to determine protein levels using the Bradford assay (Bio-Rad). Proteins were separated by 10% SDS-PAGE and transferred to poly-vinylidene difluoride transfer membranes. The blots were blocked with freshly prepared 5% non-fat milk in PBST for 2 h at room temperature. Then the blots were incubated with specific primary antibodies overnight at 4°C. HRP-conjugated secondary antibodies and ECL substrate (CLiNX) were used for detection.

### Immunohistochemistry

For IHC staining, the selected tumor tissue blocks were cut into 4-μm-thick sections on SuperFrost Plus slides (Thermo Fisher Scientific). Antigen retrieval was achieved by heating the slides in 0.01 M citrate buffer (pH 6.0) in a microwave for 20 min. Endogenous peroxidase activity was blocked using 3% H_2_O_2_. Primary antibodies against human FGF18 were applied.

Antigen visualization was performed using the Thermo Scientific UltraVision LP Detection System (United States). Immunohistochemical reactions were developed with diaminobenzidine, and sections were counterstained with Harris hematoxylin. All immunostains were manually processed.

### *In vivo* Mice Model

Female BALB/c nude mice (4 weeks old) were obtained from SLACOM and used as xenograft models. Nude mice were maintained under pathogen-free conditions and treated with the approval of the Animal Care Committee of Fudan University. 1 × 10^6^ cells transfected with NC or FGF18 were suspended in 0.2 mL of PBS and subcutaneously injected (*n* = 5). Tumor volume was calculated every 5 days. After 30 days, mice were sacrificed and xenograft tumors were sent for further validation.

For metastasis analyses, we transfected luciferase expression vectors into both NC and FGF18 overexpressed 786-O cells. Cells were intravenously injected into mice tails. After 30 days, we analyzed 786-O cell metastasis by bioluminescence imaging with intravenous luciferin injection (150 mg D-luciferin/kg body weight) into tails of mice.

### Statistical Analysis

All experiments were repeated at least three times. Statistical analyses were performed only when a minimum of *n* = 3 independent samples were acquired. All data were expressed as mean ± SD. Statistical analysis was performed with GraphPad Prism 6.0 software (GraphPad).

## Results

### Differential FGF18 Expression Value in Paired Normal and Tumor Tissue in Different Cancer Types

**FIGURE 1:**
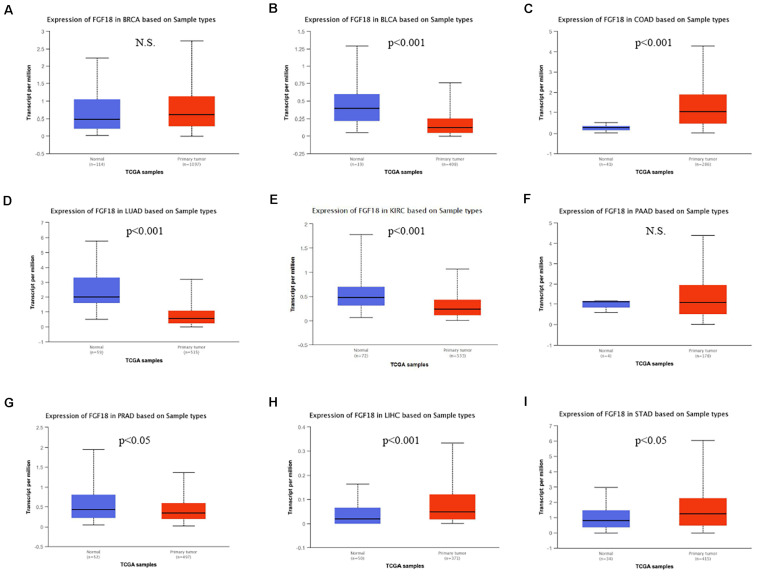
Differential expression of FGF18 in several cancer types based on the TCGA database. Expression levels of FGF18 between normal and primary tumor samples from TCGA were investigated, which included **(A)** breast invasive carcinoma (BRCA), **(B)** bladder urothelial carcinoma (BLCA), **(C)** colon adenocarcinoma (COAD), **(D)** lung adenocarcinoma (LUAD), **(E)** kidney renal clear cell carcinoma (KIRC), **(F)** pancreatic adenocarcinoma (PAAD), **(G)** prostate adenocarcinoma (PRAD), **(H)** liver hepatocellular carcinoma (LIHC), and **(I)** stomach adenocarcinoma (STAD) (N.S., No statistical significance).

Fibroblast growth factor 18 was thought to be a tumor promotor and highly expressed in tumor tissue compared to paired normal tissue in various tumor types. To determine FGF18 expression in different tumors, we investigated FGF18 expression in different paired normal and cancer tissues, namely BRCA (Figure 1A), BLCA (Figure 1B), COAD (Figure 1C), LUAD (Figure 1D), KIRC (Figure 1E), PAAD (Figure 1F), PRAD (Figure 1G), LIHC (Figure 1H), and STAD (Figure 1I), using the UALCAN database from TCGA database. Surprisingly, FGF18 were expressed significantly lower in the cancer tissue of KIRC, LUAD, BLCA, and PRAD compared to normal tissues, while FGF18 were significantly up-regulated in the cancer tissue of COAD, LIHC, and STAD, compared to normal tissues.

### FGF18 Expression Correlates With Clinicopathological Characteristics of Clinical ccRCC Tissues

**FIGURE 2:**
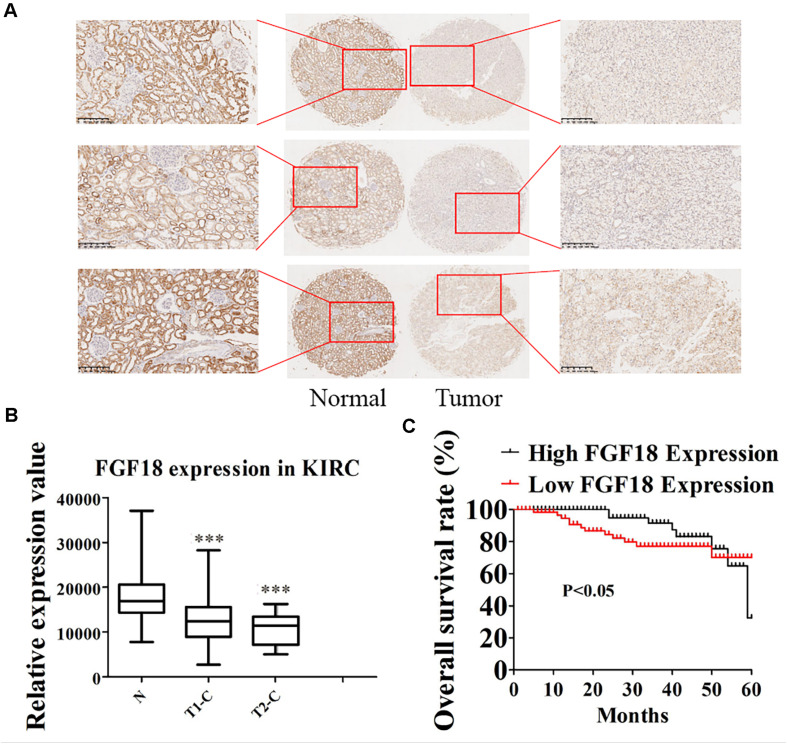
Low expression of FGF18 in ccRCC correlates with better patient prognosis. **(A)** The expression of FGF18 in ccRCC was analyzed using IHC on a ccRCC tissue microarray (82 cases). **(B)** Analysis of relatively FGF18 expression in ccRCC normal tissue, cancer tissue of T1 stage, and cancer tissue of the T2 stage. ^∗∗∗^*p* < 0.001 versus the normal group. **(C)** The prognostic significance of FGF18 expression for ccRCC patients was determined with IHC values, using the median value as the cutoff. The observation time was 60 months.

**FIGURE 3:**
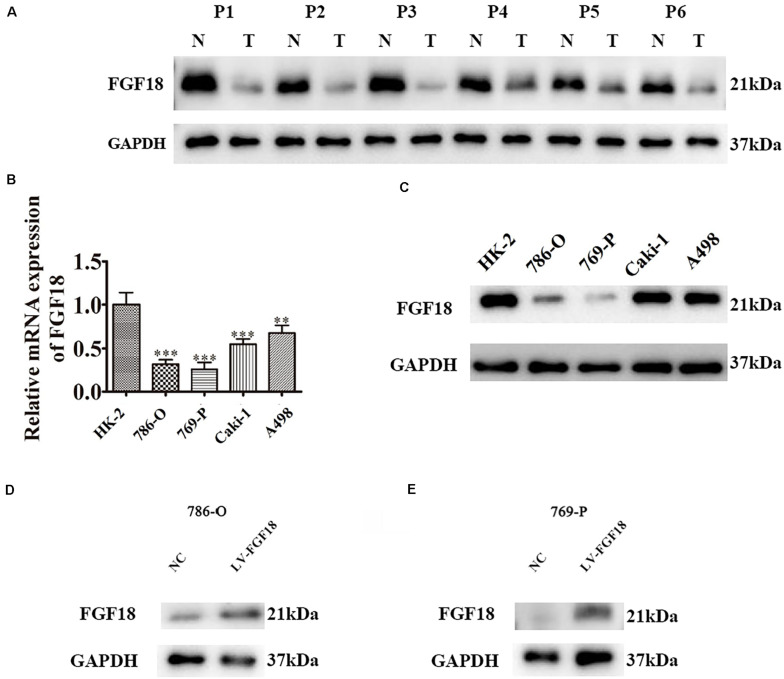
Low expression of FGF18 in ccRCC clinical samples and cell lines. **(A)** The expression of FGF18 in six paired ccRCC normal and tumor tissues were analyzed with western blot. N, normal tissues; T, tumor tissues. **(B)** FGF18 expression in 786-O, 769-P, Caki-1, A498, and the normal renal epithelial cells HK-2 with RT-PCR. Data of three experiments are presented as the mean ± SD. ^∗∗^*P* < 0.01, ^∗∗∗^*P* < 0.001 versus the normal group. **(C)** FGF18 expression in 786-O, 769-P, Caki-1, A498, and the normal renal epithelial cells HK-2 with western blot. **(D)** Overexpression of FGF18 in 786-O using lentivirus. **(E)** Overexpression of FGF18 in 769-P using lentivirus.

We began to validate the FGF18 expression in paired normal and cancer tissues of 82 ccRCC patients enrolled between 2008 and 2014 in Huashan Hospital with tissue microarray (Table 1). Representative examples of expression of FGF18 are shown in Figure 2. In the non-neoplastic renal tissue of ccRCC, the nephric tubules showed strong FGF18 immunoreactivity but glomerular epithelial cells and pelvic urothelium lacked FGF18 immunoreactivity (Figure 2A). In paired ccRCC neoplastic tissue, the ccRCC tumor tissue showed relatively low FGF18 immunoreactivity. We further validated higher FGF18 expression in normal renal tissue than paired ccRCC tumor tissues in five paired non-neoplastic and neoplastic tissues (Figure 3A).

**TABLE 1:** The clinic-pathological factors of 82 KIRC patients.

Characteristics	Numbers	FGF18		*P*-value
		Low (*N* = 39)	High (*N* = 43)	
**Sex**				0.875
Male	46	21	25	
Female	36	18	18	
**Age**				0.683
≤60	35	16	19	
>60	47	23	24	
**TNM stage**				0.045
I	72	32	40	
II	10	7	3	
**Tumor size**				0.011
≤3 cm	33	12	21	
>3 cm	49	27	22	

*NS, no statistical difference.*

Fibroblast growth factor 18 expression and the clinicopathological data of ccRCC patients are summarized in Table 1. FGF18 expressions were not associated with patient age (*p* = 0.683, N.S.) or gender (*p* = 0.875, N.S.). As for tumor grade, high FGF18 expression was noted in 40 of 72 ccRCC T1 cases (55%) and in 3 of 10 ccRCC T2 cases (30%), which indicates that high ccRCC tumor grades correlate with low FGF18 expression (*p* = 0.045 < 0.05) (Figure 2B). Furthermore, FGF18 expression significantly decreased with increasing tumor size (*P* = 0.011 < 0.05), as high FGF18 expression was noted in 22 of 49 ccRCC tumor size >3 cm (45%) cases and 21 of 33 ccRCC tumor size ≤3 cm (64%) cases. Kaplan-Meier survival curves showed that high FGF18 expression was related to good survival with statistical significance compared to low FGF18 expression (*p* = 0.042 < 0.05) (Figure 2C).

### Overexpression of FGF18 Inhibits ccRCC Cell Proliferation and Invasion *in vitro* and *in vivo*

**FIGURE 4:**
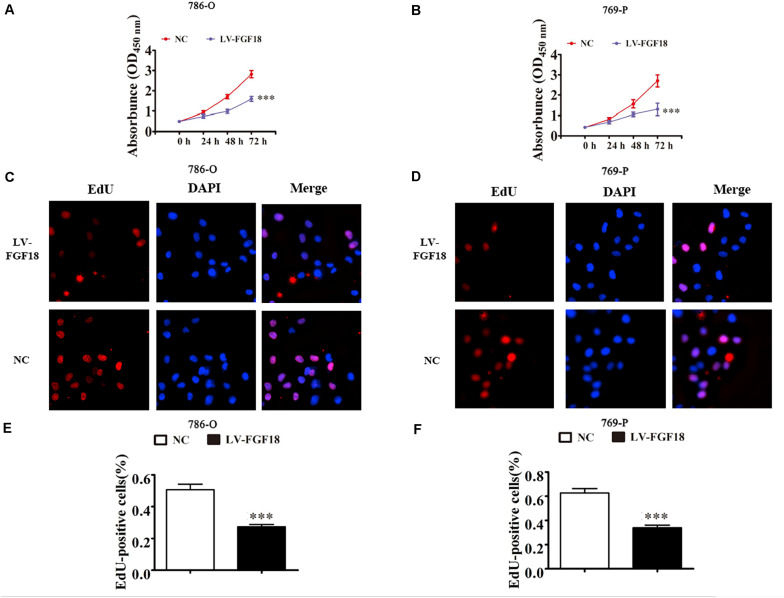
FGF18 overexpression suppresses cellular proliferation in ccRCC cells. **(A,B)** CCK8 assay showed that FGF18 overexpression reduced the proliferation of both 786-O and 769-P cells. Data of three experiments are presented as the mean ± SD. ^∗∗∗^*p* < 0.001 versus NC. **(C,D)** EdU assay compared proliferation ability between FGF18 over-expression and NC group in both 786-O and 769-P cells. **(E,F)** The relative percentage of EdU positive cells was calculated for both 786-O and 769-P cells in three experiments. Data of three experiments are presented as the mean ± SD, ^∗∗∗^*p* < 0.001 versus NC.

**FIGURE 5:**
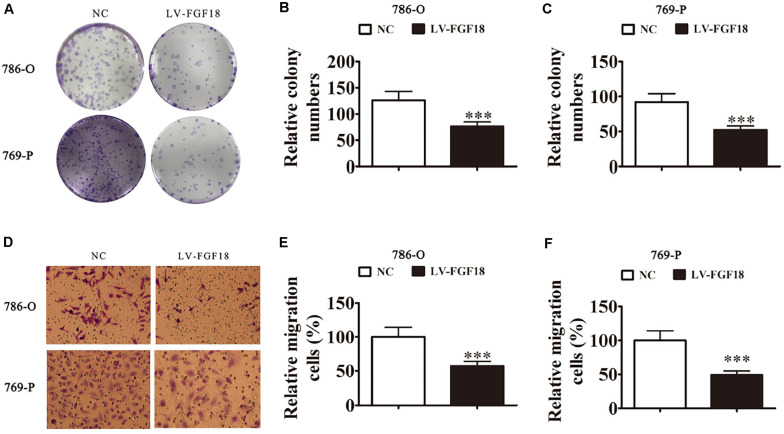
FGF18 overexpression suppresses cellular invasion in ccRCC cells. **(A)** Cloning formation assays showed that the overexpression of FGF18 reduced the proliferation of both 786-O and 769-P cells. **(B,C)** The relative cloning numbers were calculated of both 786-O and 769-P cells. **(D–F)** Transwell assay showed that FGF18 overexpression reduced the migration ability of both 786-O and 769-P cells. Data of three experiments are presented as the mean ± SD, ^∗∗∗^*p* < 0.001 versus NC.

**FIGURE 6:**
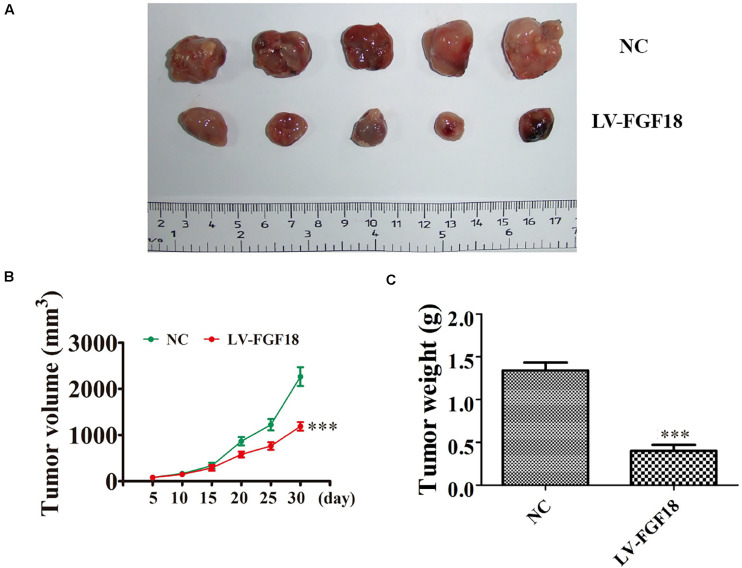
*In vivo* xenograft mice model validates FGF18 as a tumor suppressor in ccRCC cell. **(A)** Representative photographs of 786-O tumor formation in the xenograft nude mice model. **(B)** Summary of the tumor volume in mice that were measured weekly. Data are presented as mean ± SD. ^∗∗∗^*p* < 0.001 versus NC. **(C)** Tumor weight was measured 30 days post-injection. Data are presented as mean ± SD, ^∗∗∗^*p* < 0.001 versus NC.

**FIGURE 7:**
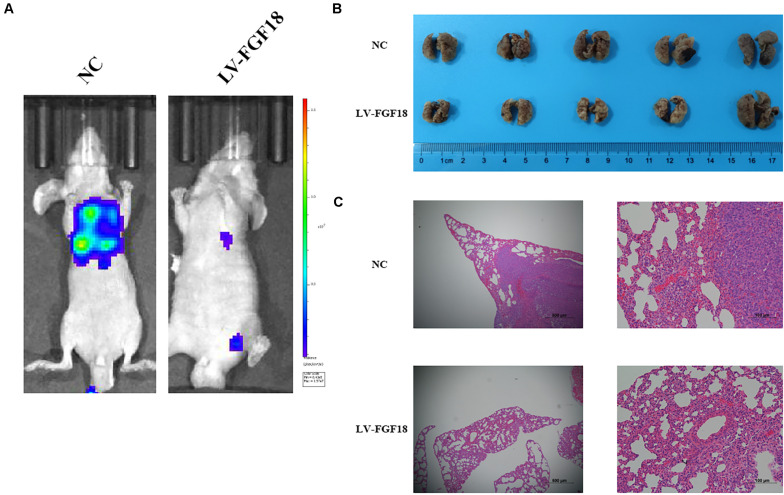
Overexpression of FGF18 inhibits the metastasis ability of ccRCC cell *in vivo*. **(A)** Live imaging showed the effect of overexpression of FGF18 on metastasis of 786-O-NC-luc cells or 786-O-FGF18-luc cells 30 days after tail injection. **(B)** Representative photographs of lung metastasis of nude mice (*n* = 5). **(C)** HE staining of lung metastasis sites of 786-O-NC-luc cells or 786-O-FGF18-luc cells in the nude mice metastasis model.

We further determined the levels of FGF18 expression in ccRCC cell lines and normal renal tubular epithelial cell lines. Expression of FGF18 was much lower at both mRNA (Figure 3B) and protein levels (Figure 3C) in 786-O, Caki-1,769-P, and A498 than in HK-2. To determine the biological effect FGF18 exerted on ccRCC cells, we employed lentivirus to increase FGF18 expression in 786-O (Figure 3D) and 769-P cells (Figure 3E). FGF18 over-expression in 786-O and 769-P suppressed proliferation ability markedly via CCK8 assay (Figures 4A,B), EdU assay (Figures 4C–F), and Colony formation assay (Figures 5A–C). The invasive ability (Figures 5D–F) of 786-O and 769-P cells was also markedly suppressed after FGF18 overexpression. Notably, our data demonstrated that overexpression of FGF18 significantly decreased the growth rate of 786-O xenograft *in vivo* (Figures 6A,B). After 30 days, mice were sacrificed. FGF18 overexpression in 786-O significantly suppressed tumor volume and tumor weight (*p* < 0.05) (Figure 6C). In addition, FGF18 overexpressed 786-O cells, which indicates a relatively lower metastasis ability than 786-O cell (Figures 7A,B). FGF18 overexpression tends to reduce the cancer cell colony in the HE staining of lung metastasis (Figure 7C).

### Overexpression of FGF18 Inhibits the Epithelial-Mesenchymal Transition in ccRCC

**FIGURE 8:**
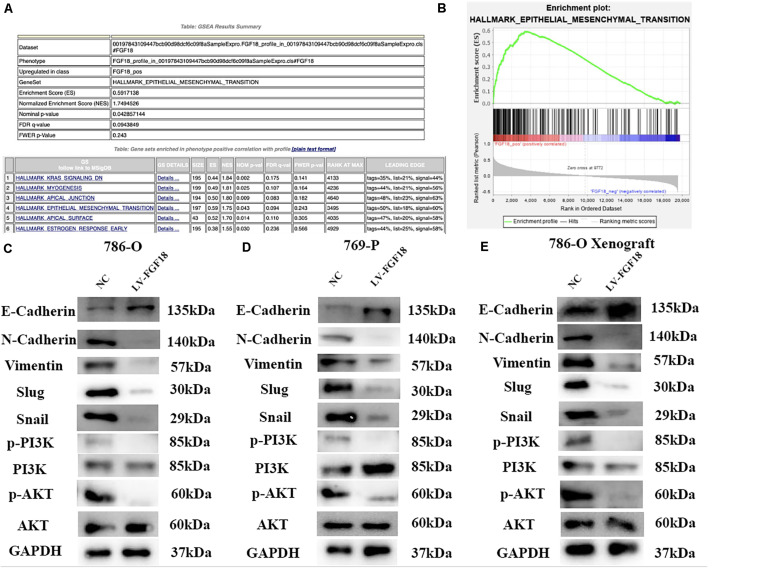
FGF18 overexpression inhibits ccRCC progression via the EMT process through PI3K/Akt signaling pathway. **(A)** GSEA was performed to identify pathways between FGF18 high expression ccRCC tissues and low expression ccRCC tissues. **(B)** EMT was involved in the FGF18 high expression group. **(C)** Western blotting analysis of protein expression of EMT and PI3K/Akt signal pathway in NC and FGF18 overexpressed 786-O cell. **(D)** Western blotting analysis of protein expression of EMT and PI3K/Akt signal pathway in NC and FGF18 overexpressed 769-P cell. **(E)** Western blotting analysis of protein expression of EMT and PI3K/Akt signal pathway in NC and FGF18 overexpressed 786-O cell xenograft mice model.

Gene set enrichment analysis (GSEA) was performed to explore the underlying mechanism of how the upregulation of FGF18 inhibits ccRCC progression. Gene profiles between high FGF18 expression group (*n* = 101) and low FGF18 expression group (*n* = 291) in the TCGA database were compared. The result revealed that several enriched pathways, including the KRAS pathway, myogenesis pathway, EMT pathway, and apical junction/surface pathway, were involved in the FGF18 high expression group (Figures 8A,B). Among these, the EMT process is closely linked to cancer cell invasion and metastasis in various types of cancers (13). Thus, we investigated the protein expression of FGF18 up-regulation on the EMT-related markers and related transcription factors in 786-O and 769-P cells. The protein levels of E-cadherin were increased while those of N-cadherin and Vimentin were decreased in FGF18 overexpressed 786-O cells compared with the control cells (Figure 8C). The expression of EMT related transcription factors in 786-O cells was also significantly inhibited after FGF18 overexpression. Similar results were observed in 769-P cells (Figure 8D). Further protein analysis of FGF18 overexpressed 786-O xenograft tumor compared to NC 786-O xenograft tumor also indicated elevated E-cadherin expression, decreased N-cadherin, Vimentin, and EMT related transcription factors (Figure 8E). Our findings demonstrated that FGF18 upregulation suppressed EMT in ccRCC cells.

### PI3K/Akt Signaling Pathway Involves in FGF18 Overexpression on the EMT Process of ccRCC

**FIGURE 9:**
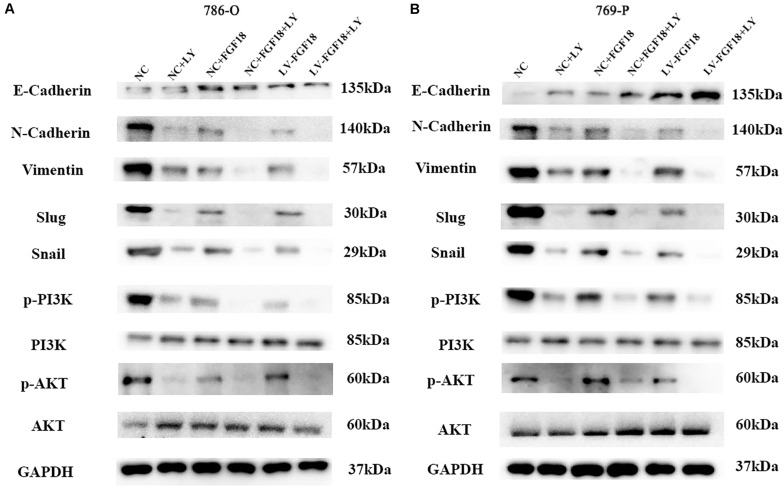
Inactivation of the PI3K/AKT pathway enhances the EMT inhibitory effect of FGF18 in ccRCC cell lines. **(A)** 786-O cells treated with LY294002 (10 μM) or recombinant FGF18 (100 ng/ml) and FGF18 overexpressed 786-O cells treated with LY294002 were incubated for 12 h for WB analyze. **(B)** 769-P cells treated with LY294002 (10 μM) or recombinant FGF18 (100 ng/ml) and FGF18 overexpressed 786-O cells treated with LY294002 were incubated for 12 h for WB analyze.

The PI3K/Akt signaling pathway, regulated by EMT, plays an important role in the biological characteristics of the cancer cell (14). Taking this into account, we speculate that FGF18 up-regulation inhibits RCC cell proliferation, invasion, and EMT via the PI3K/Akt pathway. The western blot showed that FGF18 overexpression significantly reduced p-PI3K and p-Akt expression in both 786-O cells/xenograft and 769-P cells (Figures 8C–E). To further validate the role of FGF18 in the EMT process regulated via the PI3K/Akt pathway, we treated 786-O cells with a specific PI3K inhibitor LY294002. Our results showed that overexpression of FGF18 or incubating cells with recombinant FGF18 both inhibited the EMT process as well as PI3K/Akt pathway, while LY294002 showed a synergism effect with FGF18 overexpression in 786-O cells (Figure 9A) and 769-P cells (Figure 9B). This validates the theory that FGF18 overexpression inhibits the PI3K/Akt signaling pathway and EMT process in ccRCC.

## Discussion

In recent years there has been great progress and invention in therapeutic procedures for ccRCC, but there are still some remaining limitations connected with high toxicity and non-durable response (15). Thus, there is an urgent need to discover novel targets for the prevention and treatment of ccRCC.

FGFs are growth factors secreted by cancer cells or surrounding stromal cells, and the dysregulation of the FGF signaling pathway plays an important role in the tumor microenvironment (16). FGF18 overexpression has been considered a potential prognostic biomarker in several types of tumors including hepatocellular cancers, colorectal cancer, and ovarian cancer. In ccRCC, the role of FGF18 remains unknown. In this study, we determined the differential expression of FGF18 in several cancer types in TGCA databases. We found that FGF18 was overexpressed in paired normal renal tissues versus ccRCC tissues as well as in normal renal tubular epithelial cell lines versus ccRCC cell lines and that it correlated with malignant stage level and prognosis in ccRCC patients. Further *in vitro* and *in vivo* experiments also confirmed that overexpression of FGF18 in ccRCC cell lines leads to a decrease of cell invasion and proliferation. These results indicate that overexpression of FGF18 may exhibit tumor-inhibiting effects on ccRCC.

In order to further determine the function of FGF18 in ccRCC, we performed gene set enrichment analysis based on online clinical data from the TCGA database, which revealed that the EMT pathway is involved in the FGF18 high expression group. The growing significance of EMT in the tumorigenesis of various cancers has been the subject of studies these years, and the main features comprise a loss of the epithelial markers E-cadherin and increase of the mesenchymal markers N-cadherin and Vimentin (17). EMT contributes to the increase of cell invasiveness and promotion of distant metastasis during tumorigenesis (18) and provides a promising potential strategy for cancer therapy.

In this study, we found that overexpression of FGF18 caused increased expression of E-cadherin and decreased expression N-cadherin and Vimentin in ccRCC cells, which indicated that the overexpression of FGF18 inhibits EMT in ccRCC cells. Several other studies have revealed that EMT has a close relationship with the PI3K/Akt pathway (19). We suggest that FGF18 is a potential molecular target for ccRCC treatment and that overexpression of FGF18 inhibited EMT via the PI3K/Akt pathway in ccRCC.

## Data Availability Statement

All datasets generated for this study are included in the article/supplementary material.

## Ethics Statement

The studies involving human participants were reviewed and approved by the Ethics Committee of Huashan Hospital. The patients/participants provided their written informed consent to participate in this study. The animal study was reviewed and approved by Animal Experiment Center of Pharmacodynamic Evaluation, School of Pharmacy, Fudan University.

## Author Contributions

CY, HJ, LZ, and JH designed the experiments and offered direction for the project. CY, ZZ, FY, ZM, XC, YO, CX, SW, and ZC conducted the experiments, analyzed the results, and performed bioinformatics analysis. ZZ drafted the manuscript. CY and HJ reviewed the manuscript and made revisions. All authors participated and approved the final version of the manuscript.

## Conflict of Interest

The authors declare that the research was conducted in the absence of any commercial or financial relationships that could be construed as a potential conflict of interest.

## Footnotes

1 http://ualcan.path.uab.edu/index.html
